# A nomogram to predict stricture-free survival in patients with ureteral stricture after balloon dilation

**DOI:** 10.1186/s12894-021-00896-3

**Published:** 2021-09-16

**Authors:** Jintao Hu, Cong Lai, Mingchao Gao, Kaiwen Li, Wang He, Dingjun Zhu, Wenlian Xie, Haihua Wu, Meijuan Xu, Jian Huang, Jinli Han

**Affiliations:** 1 Department of Urology, Sun Yat-Sen Memorial Hospital, Sun Yat-Sen University, 107 West Yanjiang Road, Guangzhou, 510210 China; 2 Guangdong Provincial Clinical Research Center for Urological Diseases, Guangzhou, China

**Keywords:** Nomograms, Prognosis, Recurrence, Ureteral stricture, Balloon dilation

## Abstract

**Background:**

Balloon dilation is a commonly used minimally invasive endourological treatment of ureteral stricture, but the postoperative recurrence rate is relatively high. And factors contributing to recurrence after treatment are poorly understood. Herein, we sought to develop a novel clinical nomogram to predict ureteral stricture-free survival in patients suffering from ureter stricture and performed balloon dilation.

**Methods:**

The nomogram was established based on a retrospective analysis of 321 patients who received endoscopic balloon dilation alone for ureter strictures from January 2016 to January 2020 in Sun Yat-sen Memorial Hospital using the Cox regression model. Perioperative clinical data and disease outcomes were analyzed. The primary endpoint was the onset of ureteral re-stricture after ureter balloon dilation. Discrimination of the nomogram was assessed by the concordance index (C-index) and the calibration curve. The results were internally validated using bootstrap resampling.

**Results:**

Overall, 321 patients with a median follow-up of 590 days were enrolled in the study, among which 97 patients (30.2%) developed recurrence of ureteral stricture during follow-up. Five variables remained significant predictors of ureteral re-stricture after multivariable analyses: stricture nature (P < 0.001), urinary nitrite (P = 0.041), CKD (P = 0.005), stent retention time (P < 0.001), and balloon size (P = 0.029). The calibration craves for the probability of 1-, 3-, and 5-years stricture-free survival (SFS) presented satisfied with the consistency of nomogram prediction and actual observation. The C-index of the model was 0.74 (95% CI 0.70–0.79).

**Conclusions:**

Our study developed the first nomogram to effectively predict stricture-free survival in patients suffering from ureter stricture after balloon dilation. It is helpful to identify the optimal patients with ureter stricture for balloon dilation and improve treatment outcomes. However, further external validation of the nomogram is warranted.

**Supplementary Information:**

The online version contains supplementary material available at 10.1186/s12894-021-00896-3.

## Background

Ureteral stenosis is a well-known long-term complication that can impair kidney function, mainly caused by various factors, including benign or malignant [1]. Benign ureteral stricture occurred mainly after pelvic and abdominal surgery, ureteral injuries from lithiasis and endoscopic operation, infections, retroperitoneal fibrosis, renal transplantation, urinary diversion, endometriosis, neurogenic bladder disorder, and idiopathic causes [2–4]. Urothelial carcinoma, prostatic, ovarian, metastatic cervical, breast, and colorectal cancer are the primary causes of another type of ureteral stricture: malignant ureteral stenosis [5]. Open surgery is traditionally used in ureteral reconstruction surgery. With the development of minimally invasive surgery, there have been uprising cases for pure-laparoscopic and robotic-assisted surgeries for the ureteral reconstruction. But endourological procedures are often used as a first-line treatment [6, 7]. The advantageous technique is considered a safe alternative to ureteral stricture. It postpones or even potentially shuns the needs of open or laparoscopic ureteral reimplantation [8]. Besides, many documents prove that endoscopic interventions are associated with less-invasive and cost-effective [9]. Endoscopic treatments commonly used for ureteral strictures include balloon dilation and endo-ureterectomy. Balloon dilation has been applied to treat patients with ureteral stenosis for nearly 100 years. However, little is known about the long-term results of patients undergoing balloon dilation. There is still no guidelines or consensus on balloon size, dilation duration, and postoperative double-J-stent retention time and number. According to available reports, success rates of ureteral strictures with balloon dilation vary between 33 and 100% amongst all populations [10]. Many factors may affect the success rates: aetiology, stricture location, stricture length, dilation duration, stent duration, stent number, perioperative renal function, infection. In this study, we analyzed these predictive factors for the recurrence of stricture after balloon dilation and the incidence of recurrence of stricture after dilation. We aimed to build a useful and practical nomogram for predicting the stricture recurrence of patients with ureteral stricture after ureter balloon dilation.

## Methods

### Patient and study design

We developed the nomogram from a patient cohort from Sun Yat-sen Memorial Hospital (Guangzhou, China). We utilized a retrospective cohort study of patients diagnosed with ureteral stricture from January 2016 to January 2020.

Patients fulfilling the following eligibility criteria were enrolled for analysis: (a) individuals diagnosed with ureteral strictures confirmed by imaging and ureteroscopy. The patients received imaging checkups including ultrasound, renal scintigraphy, intravenous urography, antegrade urography, retrograde urography, computed tomography urography (CTU), or magnetic resonance urography (MRU). (b) All procedures must meet treatment with balloon dilatation alone and technical success. Technical success has defined that the ureteroscope and guidewire can pass through the ureteral stricture after balloon dilated, and then successfully indwell the double J ureteral stent. (c) Patients undergoing endoscopic balloon dilation with the postoperatively indwelling ureteral stent. Patients were excluded from analysis if they had malignant tumours that were not cured or in remission or lost follow-up.

### Technique

The patient was placed in the lithotomy position or modified supine Valdivia position under local or general anaesthesia. First, retrograde ureteroscopy was used to explore the ureteral stricture, and the guidewire was inserted through the stenosis into the renal pelvis. If it failed, ultrasound-guided percutaneous nephrostomy was performed, and the guidewire was inserted into the bladder antegrade. A suitable dilatation balloon was placed into the stricture along the guidewire. Generally, according to the anatomical diameter of the ureter and clinical experience, the F24-30 balloon was selected for upper ureteral stricture, the F21-24 balloon for the middle part, and the F21 balloon for the lower segment. Under the monitoring of a ureteroscope, the balloon was filled with saline to 30 atmospheres, and the dilation time of most patients was 5 min because no better results achieved for a longer time in our preliminary trial (from 3 to 30 min) so that the stricture completely dilated without rupture of the ureter. The criteria for full dilation was that after balloon dilation, the F9.8 ureteroscope passed through the stenosis smoothly without resistance. If the first dilation didn't meet the goal, it could be dilated again or replaced with a larger balloon. After successful dilation, one or two double J stents were inserted into the ureter along the guidewire. The ureteral stent was removed after different indwelling times.

### Data collection

The following demographic and clinical characteristics had extracted from medical records: age, gender, disease characteristics, dilated side, stenosis location, aetiology classification, degree of hydronephrosis, retention time and the number of stents, balloon size, dilation duration, perioperative urine leukocyte nitrite, urine culture, Scr, and so on. When processing the data, we converted part of continuous variables into categorical variables.

All participants underwent telephone follow-up and outpatient review. Follow-up data obtained from the patient's last outpatient review and contact include laboratory examinations, imaging checkups, and recorded urological symptoms available. Stricture-free had defined as a situation that the patient had a stable renal function, no urological symptoms, no evidence of worsening hydronephrosis, and no need for further treatment after the stent was removed. Patients' stricture-free survival (SFS) was from the day of withdrawing double-J-stent to the date of relapse or last contact. The overall follow-up time had defined as the interval from surgery to recurrence or the last contact. Our primary endpoint was the recurrence status of ureteral stricture and SFS time. Finally, we established a prediction model of SFS.

### Statistical analysis

The primary endpoint was the onset of ureteral re-stricture after ureter balloon dilation. Log-rank test and Breslow test were used to find potentially independent prognostic variables. The missing baseline data were accounted for via multiple imputations, Cox regression analysis was used for multivariable analyses. The final model was determined by the forward LR step-down process that including all significant covariates from the univariate analysis. Statistical analysis to recognize independent prognostic factors was performed using SPSS version 25.0 for Windows (IBM Corporation, Armonk, New York, USA). On all tests, a p-value of < 0.05 demonstrated have a significant statistical difference. A nomogram was then developed based on the final model using the survival, RMS, and foreign package in R (version 4.0.2, R Foundation for Statistical Computing, Vienna, Austria) (http://www.R-project.org) [11].

The performance of the nomogram had assessed by discrimination and calibration. The bias of the nomogram had measured by the bootstrap bias-corrected estimate of concordance index (C-index) [12–14]. AUC was used to assess the specificity and sensitivity of the nomogram. And a bootstrapping-based calibration plot of the model had evaluated by comparing actual SFS probability with nomogram-predicted SFS probability [15]. We repeatedly fitted the nomogram to 2,000 bootstrap samples and assessed its performance on the original samples [16].

## Results

**Fig. 1:**
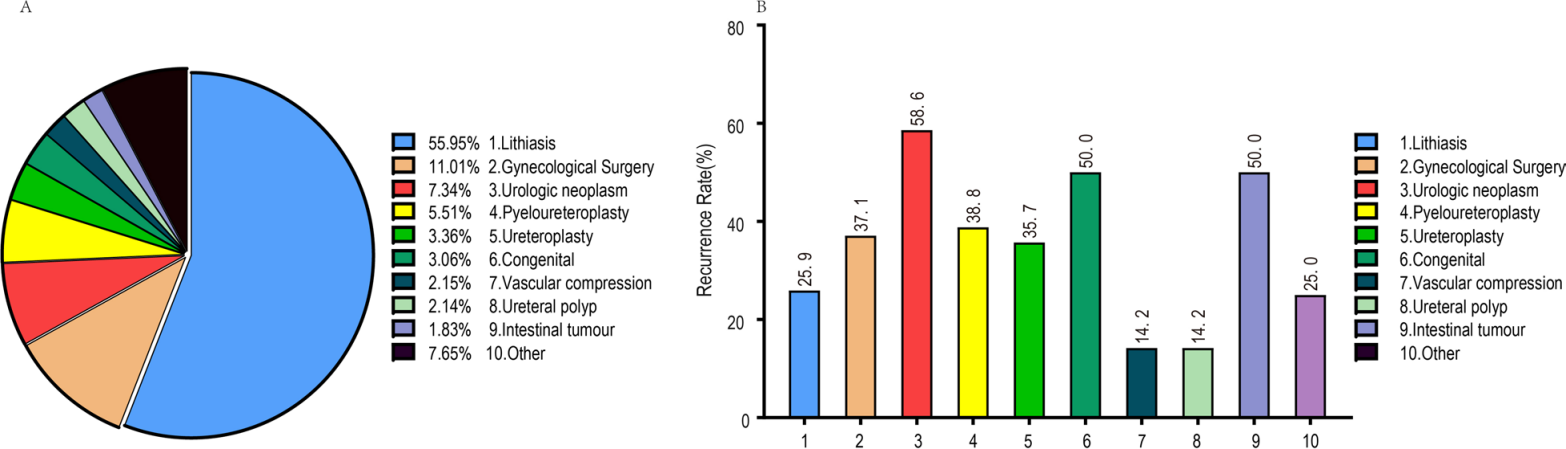
**A** Etiological Classification, **B** results of dilation according to etiological

Additional file 1: Table 1 lists the demographic and clinical characteristics. 327patients met the screening criteria of the current study. A total of 321 patients were included and analyzed due to 6 cases lost to follow-up. The ureteral stricture was most commonly secondary to ureteric lithiasis (55.96%), followed by gynaecological neoplasia surgery (11.01%) and urologic neoplasm (7.34%) (Fig. 1A). 85.02% of the ureteral stricture were benign. According to aetiology, treatment results of the obstruction have presented in Fig. 1B. During a median follow-up of 590 days, 97 of 321 patients (30.2%) developed ureteral restenosis (interquartile range (IQR) 335–1024 days). The median SFS was 406 days (IQR 93–852 days).

**Table 1:** Univariate and multivariate Cox analyses of patients with stricture recurrence after balloon dilation for stricture

Variable	Univariate Analysis			Multivariate Analysis		
	HR	95% CI	*P value*	HR	95% CI	*P value*
Nature of strictures			< 0.001			< 0.001
Benignant	1(ref)			1(ref)		
Malignant	2.558	1.617–4.046	< 0.001	3.044	2.457–3.771	< 0.001
M–S hydronephrosis			0.017			
No	1(ref)					
Yes	1.657	1.090–2.519	0.018			
Urinary nitrite (NIT)			0.020			0.041
Negative	1(ref)			1(ref)		
Positive	2.146	1.195–3.853	0.011	1.915	1.489–2.461	< 0.001
Urinary culture bacteria			0.043			
Negative	1(ref)					
Positive	1.882	1.064–3.327	0.030			
CKD			0.001			0.005
1	1(ref)		< 0.001	1(ref)		
2	1.140	0.523–2.487	0.742	1.015	0.734–1.398	0.936
3	2.055	0.953–4.432	0.066	1.460	0.966–2.206	0.006
4	2.458	0.947–6.384	0.065	1.561	1.135–2.148	0.072
5	5.021	1.975–12.764	0.001	4.120	2.806–6.049	< 0.001
Urine leukocyte			0.035			
Negative	1(ref)					
Positive	1.722	1.007–2.945	0.047			
Approach			0.028			
Retrgrade	1(ref)		0.028			
Antegrade	2.414	0.981–5.941	0.055			
Location of stenosis			0.194			
Proximal	1(ref)		0.224			
Mid	0.592	0.325–1.080	0.087			
Distal	0.854	0.535–1.363	0.508			
Balloon size			0.044			0.029
F18	1(ref)		0.035	1(ref)		
F21	0.677	0.235–1.953	0.471	0.943	0.606–1.469	0.796
F24	0.654	0.230–1.859	0.426	0.888	0.577–1.367	0.590
F30	1.273	0.448–3.613	0.650	1.814	1.164–2.827	0.008
Dilatation time			0.026			
< 5 min	1(ref)		0.034			
5–10 min	0.573	0.324–1.014	0.056			
> 10 min	1.340	0.802–2.239	0.263			
Operation side per patient			0.504			
Left	1(ref)					
Right	0.873	0.586–1.301	0.504			
Stent retention time			< 0.001			< 0.001
< 3 months	1(ref)		< 0.001	1(ref)		
3–6 months	0.996	0.584–1.700	0.989	1.13	0.902–1.416	0.289
> 6 months	3.302	2.015–5.412	< 0.001	3.043	2.455–3.772	< 0.001
Stent retention number			0.291			
1	1(ref)					
2	1.243	0.829–1.864	0.293			

*M–S hydronephrosis* moderate severe hydronephrosis, *CKD* chronic kidney disease, *CI* confidence intervals

The overall ureteral re-stricture rate was 30.2%. Concerning etiological factors, we identified that patients who took radical cystectomy experienced the highest ureteral restenosis rate (63.1%), followed by intestinal tumours (50%). Table 1 showed the results of the univariable and multivariable Cox regression analyses used to forecast the likelihood of stricture recurrence upon dilation surgery. After applying a forward stepwise identification process, the best-simplified nomogram had developed. We found that stricture nature (P < 0.001), urinary nitrite (P = 0.041), CKD (P = 0.005), stent retention time (P < 0.001), and balloon size (P = 0.029) were independent risk factors for relapse of ureter stricture. Patients with malignant stricture, urinary nitrite positive, and CKD 5 were prone to ureteral restenosis, while patients with stent retention time < 3 months and dilation with F24 balloon had the highest ureteral patency rate.

**Fig. 2:**
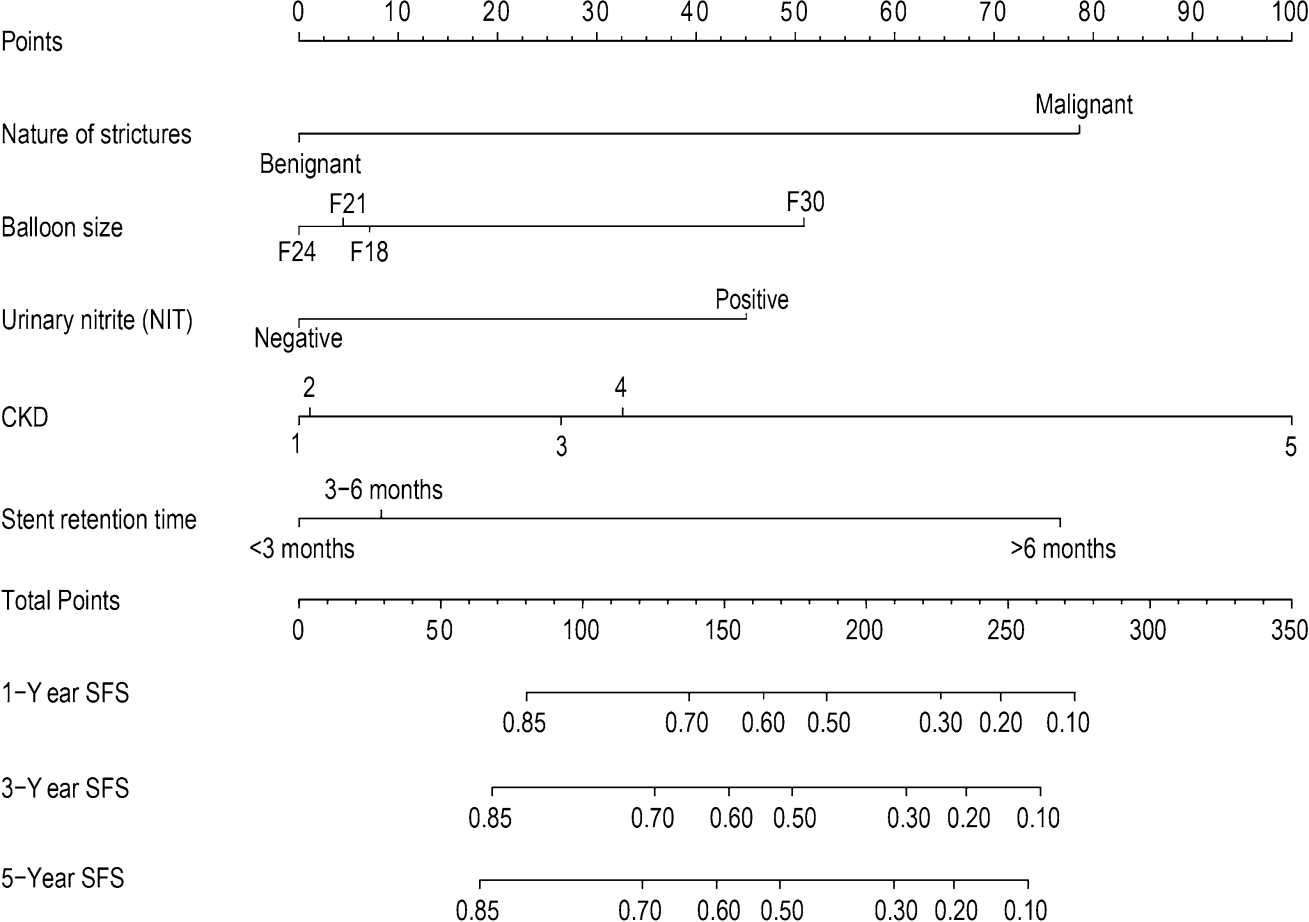
Nomogram predicting 1-, 3-, and 5-year SFS for patients after balloon dilation

**Fig. 3:**
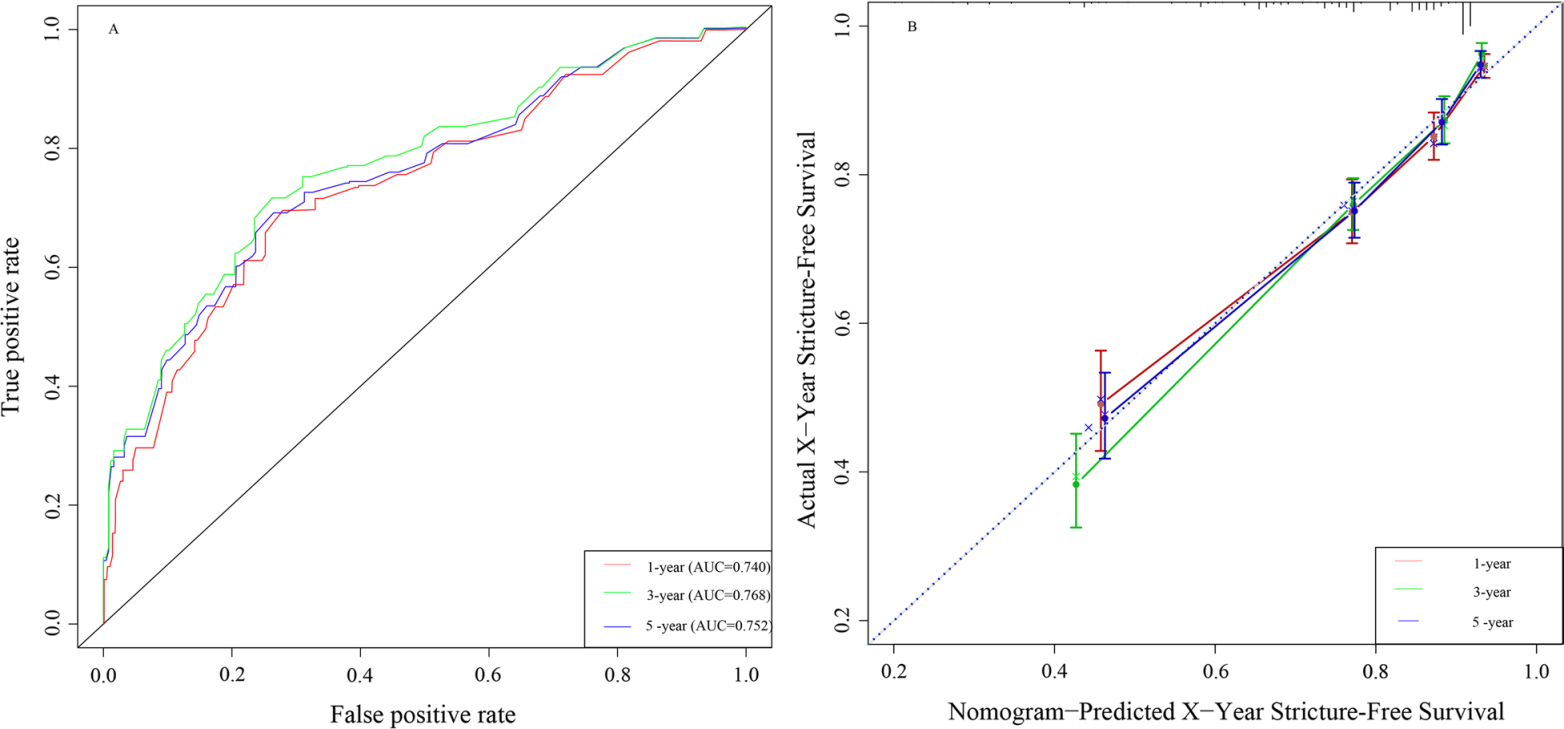
**A** Receiver operating characteristic curve analysis for evaluating the accuracy of the 1-, 3-, and 5-year nomogram. **B** The calibration plots of the nomogram for predicting 1-, 3-, and 5-year stricture-free survival. Nomogram-predicted SFS is plotted on the X-axis; actual SFS is plotted on the Y-axis

The predictive nomogram that integrated all these significant predictors for 1-year, 3-year, and 5-year SFS was constructed and had depicted in Fig. 2. The nomogram had good predictive ability with a C-index of 0.74 (95% CI 0.70–0.79). Internal validation showed that the 1-, 3-, and 5-year SFS predictive accuracy of our nomogram (measured by AUC values) was 0.740, 0.768, and 0.752, respectively (Fig. 3A). Besides, a favourable consistency had shown between the predicted and actual 1-,3-, and 5-year SFS probability, which validated the accuracy of the model as demonstrated by Fig. 3B.

## Discussions

To our knowledge, this study is the first to develop and validate a predictive nomogram to identify patients at increased risk of ureteral restenosis after balloon dilation. The model incorporated five items that should be evaluated with priority in clinical practice, including narrow nature, balloon size, NIT, CKD, and stent retention time.

In our study, 97 patients relapsed during follow-up, with the overall ureteral re-stricture rate was 30.2%. The results are superior to a patency rate of 41.1% reported by Campschroer et al. [8 17]. Similarly to Stehman, we observed that benign stenosis is more suitable for balloon dilation than malignant stenosis (recurrence rate:25.8%vs51.0%) [4 18]. As was shown in Fig. 1, the lowest recurrence rate had seen in stenosis secondary to both ureteral polyps (14.2%) and vascular compression (14.2%), followed by 142 secondaries to lithiasis (25.9%). All of them belonged to benign stricture. The long-term patency rate of dilation for benign stenosis was comparable to that of open surgery, which indicated that balloon dilation could replace open surgery to treat benign ureteral strictures. However, 14.6% of patients failed immediately after the stent was withdrawing, and a new stent was required to be inserting. These patients were considered not suitable for balloon dilation unless not tolerate open surgery. 41.67% of the above patients belonged to malignant. Therefore, the best indication for balloon expansion is benign stenosis, such as surgery secondary to stones, ureteral polyps, vascular compression, and benign gynaecological diseases.

Some strictures (19 5.81%) were observed secondary to the laparoscopic radical cystectomy (LRC 12 3.67% recurrence rate:66.67%) or the robot-assisted radical cystectomy (RARC 7 2.14% recurrence rate:28.5%). These strictures were possibly secondary to ischemia in the context of a widespread incision [19]. It is worth noting that stenosis secondary to RARC has a lower recurrence rate after balloon dilation than other stenoses secondary to LRC.

In univariate analysis, no significant statistical difference had revealed among different stenosis sites or the number of stents retained. It is similar to previous studies that reported that there was no apparent association between stricture location (upper, mid, or distal ureter) with long-term success rate [18, 20–24]. M.J.van Son's study reported that the stricture side was an independent risk factor for stricture recurrence (P = 0.009, HR 0.35, 95%CI 0.16–0.77) [8]. However, there was no significant difference in SFS between patients with left ureter stricture and those with the right ureter stricture in our study (P = 0.504). Interestingly, our results found no significant difference in SFS between one and two stents retention (P = 0.293). Two stents retention might cause ureteral ischemia that leads to a bad outcome.

Stavros I. reported that when indwelling stents for prolonged periods, stents may prevent tissue repair for inflammation [17]. In our study, balloon sizes were associated independently with SFS, which is consistent with several previous studies showing that appropriate size could reduce the damage to the blood supply and rupture the fibrous tissue in the stenosis [10, 25].

CKD is also considered to be an independent risk factor for patients with stenosis recurrence after balloon dilation, which is consistent with previous studies that kidney function was considered an important predictor [17, 26]. Moreover, our study showed a significant positive correlation between the five CKD stages.

Our study possesses several strengths. First, our nomogram had established through the analysis of a relatively large patients’ cohort. Furthermore, the calculation method of follow-up time used more accurately reflected the recurrence of stenosis. Also, the nomogram is easily applicable in clinical practice. Therefore, it can use as an essential screening tool that allows us to improve clinical care decisions for patients with a ureteral stricture. Surgeons and patients could make better choices through this usable rating tool before surgery. However, our research also has some limitations. First, the developed nomogram was based on data obtained retrospectively from a single center. Second, due to missing data, some important factors such as the length and diameter of the stenosis and spilt renal function were not included in this study. Although this model showed good predictive ability through internal validation, it still needs external validation.

## Conclusion

Our study developed the first nomogram to effectively predict stricture-free survival in patients suffering from ureter stricture after balloon dilation. It is helpful to identify the optimal patients with ureter stricture for balloon dilation and improve treatment outcomes. However, further external validation of the nomogram is warranted.

## Supplementary Information

**Additional file 1: Table 1**. Baseline characteristics of the study population.

## Data Availability

The datasets analyzed during the current study available from the corresponding author on reasonable request.

## Abbreviations

US: Ureteral stricture
C-index: Concordance index
AUC: Area under the curve
SFS: Stricture-free survival
CKD: Chronic kidney disease
BMI: Body mass index
Scr: Serum creatinine
eGFR: Estimated glomerular filtration rate
SD: Standard deviation
IQR: Interquartile range
